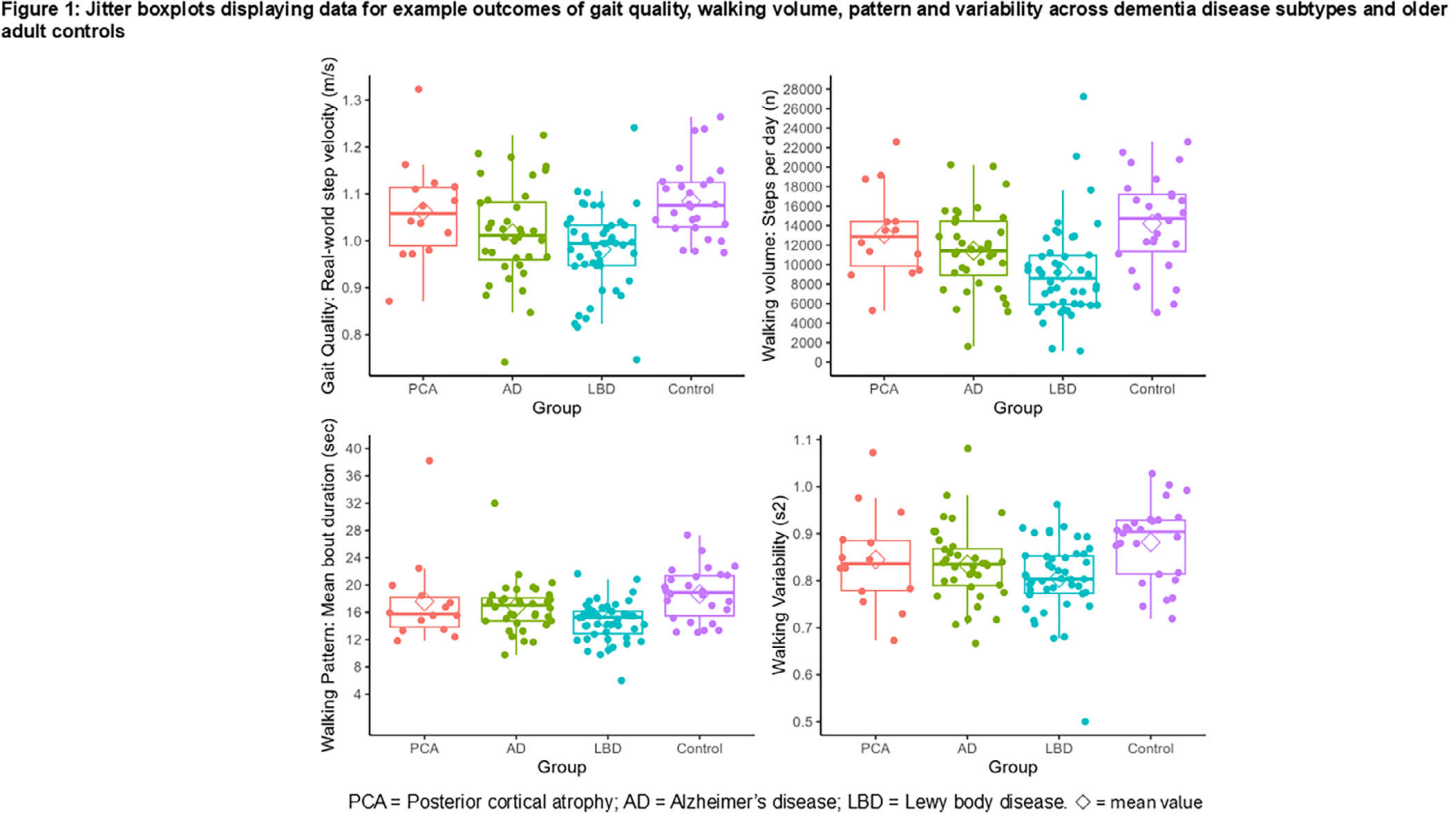# Characterising real‐world macro walking outcomes of people with Posterior Cortical Atrophy using accelerometery: Feasibility and preliminary comparison with Alzheimer's and Lewy body disease

**DOI:** 10.1002/alz70856_104769

**Published:** 2026-01-07

**Authors:** Ríona Mc Ardle, Cameron Kirk, Silvia Del Din, Yuhan Bai, Diego Kaski, Jonathan D. Rohrer, Brook Galna, Alan J Thomas, Lynn Rochester, Matthew J Bancroft, Keir X X Yong

**Affiliations:** ^1^ NIHR Newcastle Biomedical Research Centre, Newcastle University, Newcastle upon Tyne, Tyne and Wear, United Kingdom; ^2^ Translational and Clinical Research Institute, Newcastle University, Newcastle upon Tyne, Tyne and Wear, United Kingdom; ^3^ Newcastle University, Newcastle Upon Tyne, United Kingdom; ^4^ National Institute for Health and Care Research, Biomedical Research Centre, Newcastle upon Tyne, Tyne and Wear, United Kingdom; ^5^ University College London, London, United Kingdom; ^6^ Centre for Vestibular and Behavioural Neurosciences, UCL Queen Square Institute of Neurology, London, United Kingdom; ^7^ Queen Square Institute of Neurology, University College London, London, UK, London, United Kingdom; ^8^ Dementia Research Centre, Queen Square Institute of Neurology, University College London, London, ‐, United Kingdom; ^9^ Centre for Healthy Ageing, Murdoch University, Murdoch, Perth, Western Australia, Australia; ^10^ Newcastle University, Newcastle upon Tyne, Newcastle, United Kingdom; ^11^ Newcastle University, Translational And Clinical Research Institute, Newcastle upon Tyne, United Kingdom; ^12^ Dementia Research Centre, UCL Queen Square Institute of Neurology, University College London, London, United Kingdom

## Abstract

**Background:**

Posterior cortical atrophy (PCA) is a dementia subgroup commonly misdiagnosed due to unusual presentation and limited clinical awareness. Previously, the GaitDem study provided proof‐of‐concept for the use of accelerometery‐based walking assessment in clinical and real‐world settings in supporting differentiation Lewy body disease (LBD) and Alzheimer's disease (AD). Real‐world walking assessment also provides insights into the impact of disease on everyday behaviours. Here, we aimed to assess the feasibility of accelerometery‐based real‐world walking assessment of PCA and describe differences between PCA and more prevalent neurodegenerative dementia syndromes, AD and LBD.

**Methods:**

Fourteen participants with PCA (Age: 71 years(56‐78); 57% female) wore an accelerometer (AX6, Axivity) affixed to their lower back for up to seven days. Using validated algorithms, real‐world walking outcomes were derived including measures of walking quality (step velocity), volume (minutes spent walking, steps per day, bouts per day), pattern (mean bout duration) and variability (of bout durations). Data was compared to the GaitDem cohort, which included 36 people with AD (Age: 77 years(67‐88); 58% female) and 46 with LBD (Age: 77 years(65‐91); 17% female), following a similar protocol. Kruskal‐Wallis Test assessed between‐group differences with post‐hoc Dunn tests. 26 controls (Age: 74(60‐89), 58% female) were included for visual comparison (Figure 1).

**Results:**

Twelve PCA participants completed seven days of real‐world walking assessment; two completed 5‐6 days. The PCA group walked faster, spent more minutes walking, took more steps and walking bouts per day (*p* <0.01 for all) than the LBD group; no significant differences were found for pattern and variability outcomes or between PCA and AD groups (*p* >0.05; Figure 1).

**Conclusion:**

All PCA participants completed real‐world walking assessment for the recommended period of >3 days, suggesting feasibility. Preliminary results suggest that the PCA group's real‐world macro walking behaviours are more similar to AD than LBD. Despite a small sample, this novel data provides proof‐of‐concept. Only real‐world macro walking outcomes are reported. Previously, clinic‐based accelerometery outcomes relating to micro gait characteristics (e.g. spatiotemporal and signal‐based features) were more sensitive to differences between LBD and AD; this will be further explored for PCA.